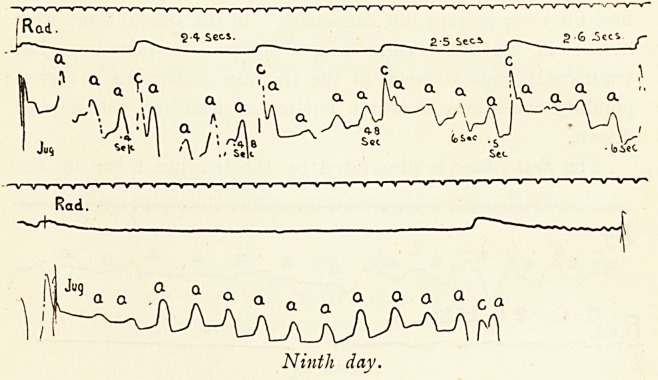# An Example of the Stokes-Adams Syndrome

**Published:** 1913-03

**Authors:** Carey Coombs

**Affiliations:** Assistant Physician to the Bristol General Hospital.


### AN EXAMPLE OF THE STOKES-ADAMS SYNDROME.

BY

Carey Coombs, M.D. Lond., M.R.C.P.,
*Assistant Physician to the Bristol General Hospital.*

This case is reported because of the interesting progress of events, especially in auriculo-ventricular block.

The patient, a maiden lady, aged 84, had rheumatic fever at 18, but no further ill-health apart from osteoarthritis and cataract. About two years before this final illness about to be described she had fallen downstairs in a mysterious and unaccountable way ; beyond this there was absolutely no hint of cardiac disease and the pulse was always regular.

About 4 p.m. on the first day of her fatal illness she was suddenly seized with alarming giddiness and fell on the floor. Seen a few minutes later, she was lying on a couch, agitated and sweating a little, the temperature normal, the pulse 120 and quite regular. She suddenly turned pale, the pulse became very slow (18 per minute) and a brief epileptiform seizure occurred. She was put to bed, and after vomiting twice passed a fairly quiet night. On the following day, when the first tracings were taken, she had attacks all day at brief intervals. The pulse showed paroxysms of irregularity corresponding to the fits.

This was succeeded by two or three days of gradual improvement, the fourth and fifth days being practically free from attacks, so that there was ground for hoping for complete recovery.

However, at 9 a.m. on the sixth day, she was, through a misunderstanding, allowed to get out of bed. The fits began again almost at once, and increased in number till her death in coma on the tenth day. Post-mortem examination was impossible.

The case, therefore, divides itself into three periods. In the first fits were present but decreasing; in the second there were none; in the third there was a progressive increase. The ventricular pauses shown in the tracings underwent a corresponding evolution, to which particular attention will now be drawn.

The *first phase* is illustrated by the tracings taken on the

*Second day.*

second day. Here a normal or practically normal auriculo-ventricular sequence was suddenly interrupted by a bout of total heart-block, in which the auricle beat at 100 per minute, and the ventricle assumed an independent rhythm at 22—23 per minute. The cessation of this paroxysm was as abrupt as its onset, and the pulse immediately afterwards became normal ($a$—$c$ = 0.2 second). The third portion of the second-day tracings shows a long ventricular pause; during this the jugular wave shows a regular auricular rhythm, together with other waves which I do not attempt to explain.

The *second phase*, that of quiescence, displays a normal auriculo-ventricular sequence, as the tracings taken on the fourth day show ; there is a definite fourth wave.

*Fourth day.*

The *third phase* of decline is well exemplified by the tracings taken on the ninth day, about twenty-four hours before death.

*Ninth day.*

At this time the fits were very frequent and the patient was in a continuously stuporose condition.

The upper strip shows a period in which there were four auricular beats to each ventricular systole. An extreme depression of conductivity is to be inferred from the fact that although only 24 to 25 stimuli passed over from auricle to ventricle per minute, even this light task was too much for the conducting system, as the increasing *a*—*c* interval shows (0.4 second, 0.48 second, 0.48 second, 0.5 second, 0.6 second). The lower strip coincided with a minor fit, and shows a ventricular pause of

8 seconds or longer, the auricular beat being regular at about 100 per minute. The longest observed pause (measured with the finger and watch) was about 30 seconds.

The other *signs of cardio-vascular disease* were scanty and relatively trivial. The arteries were thickened and tortuous, the systolic tension at first being 180 mm. Hg. There was no cardiac pain, and (apart from the attacks) no dyspnœa; the ankles were a little swollen when the patient was first seen, but this soon subsided. Two days before death the urine contained albumin, but not before. It was impossible to localise the point of maximum impulse, but the first sound, which was doubled, was heard most clearly external to the left mammary line. The patient being very stout and emphysematous, it was impossible to delimit the deep or superficial cardiac dulness. All over the præcordium, but pre-eminently at the base, there was a long, rough systolic murmur. No sounds were heard during the ventricular pauses, but the jugular beats were visible. Towards the end there were occasional extrasystoles.

The *cerebral symptoms* deserve brief notice. The fits were always produced by a ventricular pause if it attained a duration of five seconds. The sequence in a fully developed attack was as follows: the radial pulse stopped; the patient felt the attack coming, looked alarmed, and said, "I'm going;" her face turned pale, the eyes closed, and she became unconscious and rigid; cyanosis was added to pallor, and mild clonic movements of the whole body occurred, in the face first; the pulse returned, the skin became flushed and moist, and the eyes opened staringly; and she became conscious again, though a little dazed at first. Towards the end the attacks, though more frequent, were less distinct and without muscular convulsion. There were certain *mental* changes; hallucinations, which at first followed the fits at times and later became more frequent, and stupor during the last day or two, deepening eventually to coma. The *respiratory* rhythm underwent variations; at the onset of a fit there was either a heavy sigh or a series of deep breaths; and after a bout of attacks she took many deep "air-hunger" breaths which called the extraordinary muscles into action, sinking gradually to an interval of almost complete apnœa—a partial Cheyne-Stokes rhythm.

No *drug* was of real value. During the stage of improvement, it is true, atropine was given with ammonium bromide till a physiological effect was seen ; but such amelioration as occurred was due to absolute rest almost as certainly as the fatal relapse was initiated by her unfortunate getting out of bed.

*Summary.*—The case recorded is one in which various cerebral symptoms were prominent. These are to be referred to anæmia of the brain due to ventricular pauses, which again resulted from auriculo-ventricular heart-block in an arterio-sclerotic subject.

Auriculo-ventricular conductivity varied remarkably ; in the first phase it passed suddenly from the normal to complete dissociation, and as suddenly to the normal again. In the second phase it was continuously normal. The third phase was marked by a continuous and progressive decline in conductivity.

## Figures and Tables

**Figure f1:**
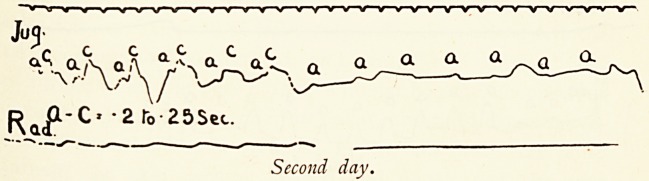


**Figure f2:**
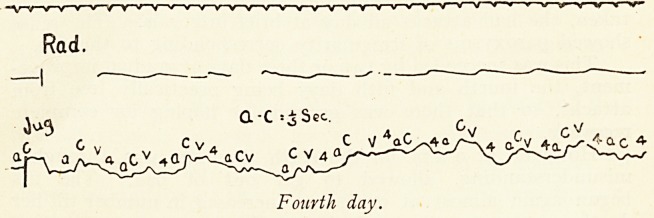


**Figure f3:**